# Simple method for confirming tibial osteotomy during total knee arthroplasty

**DOI:** 10.1186/1758-2555-4-44

**Published:** 2012-11-15

**Authors:** Hirotaka Mutsuzaki, Kotaro Ikeda

**Affiliations:** 1 Department of Orthopaedic Surgery, Ibaraki Prefectural University of Health Sciences, 4669-2 Ami Ami-machi, Inashiki-gun, Ibaraki, 300-0394, Japan; 2 Department of Orthopaedic Surgery, Ichihara Hospital, 3681 Ozone, Tsukuba, Ibaraki, 300-3295, Japan

**Keywords:** Total knee arthroplasty, Tibial osteotomy, Extramedullary, Alignment, Protractor

## Abstract

**Background:**

Achieving precise implant alignment is crucial for producing good outcomes after total knee arthroplasty (TKA). We introduce a simple method for confirming the accuracy of tibial osteotomy during TKA.

**Findings:**

Two metallic markers were placed on the skin 20 cm apart, one on the tibial tuberosity and other on the tibial crest, points that are easily identified and palpated intraoperatively. Anteroposterior radiographs of the legs were obtained. We defined the line along the markers as the tuberosity line. The osteotomy line is perpendicular to the anatomical axis of the tibia. We then calculated the angle between these two lines and designated it the osteotomy angle. We set the osteotomy angle of the protractor, and cut the bone parallel to the osteotomy line of the protractor. Postoperatively, we analyzed the varus angle of the tibial osteotomy in 35 TKAs using the protractor. The average of the varus angle of the tibial osteotomy was 89.4° ± 1.6° (95% confidence interval of −1.0976, 0.0119). There was no significant difference from the target angle of 90° (*p* = 0.055). The varus angles of 90° and 90° ± 2° for the tibial osteotomy were 42.9% and 82.9%, respectively.

**Conclusions:**

We determined the accuracy of the tibial osteotomy in the coronal plane using the protractor to be satisfactory.

## Findings

### Introduction

Correct alignment of the tibial and femoral components is one of the most important factors determining favorable long-term results of a total knee arthroplasty (TKA). It is generally accepted that the tibial component should be placed perpendicular to the anatomical axis of the tibia [1-3]. We use extramedullary guides for cutting the tibial bone because they help us avoid the potential complications of intramedullary guide use, including fat embolization, intraoperative fracture, and inability of intramedullary rod passage due to deformity [4]. Placement of the tibial component in excessive varus is the commonest cause of suboptimal alignment using extramedullary guides [5]. Coull et al. [6] showed that 48% of TKAs had tibial component angles of < 87° even when they tried to cut the tibia perpendicular to its anatomical axis. Also, there was a significant decrease in the probability if the implant was inserted in any varus alignment [7]. Aglietti and Buzzi [8] demonstrated that varus alignment of the knee and a varus tilt of more than 2° of the tibial component correlated with the incidence of lucent lines around the tibial implant. Computer-assisted navigation, digital preoperative planning and patient-specific guides improved accuracy of tibial osteotomy in TKAs [9-12]. Recently, bone-cutting devices have improved accuracy of implant positioning, even if TKA is performed manually or using minimally-invasive technique [13,14].

We developed our original intraoperative protractor to place on top of a tibial tuberosity and tibial crest so that we can easily palpate from the body’s surface intraoperatively to confirm that the tibial osteotomy has obtained the desired tibial angle. Therefore, we hypothesized that it is possible to cut a bone precisely using a protractor intraoperatively. The purpose of this study was to evaluate the accuracy of the tibial bone cut angle using an intraoperative protractor during TKA.

## Patients and methods

From October 2000 to April 2002, a total of 3 men and 24 women (aged 23–87 years, mean 61.1 years) underwent 35 TKAs for osteoarthritis (*n* = 14) or rheumatoid arthritis (*n* = 21) using the Scorpio nonrestrictive geometry cruciate retaining system (Stryker Howmedica Osteonics; Allendale, NJ, USA). The ethics committee of Ichihara Hospital reviewed and approved the study (the reference number for the ethics approval: 1203, and the trial registration number: 1203). Informed consent was obtained from each patient. A single surgeon performed all of the operations. They were done with the patient under epidural or general anesthesia and using the medial parapatellar approach. The target alignment of tibia osteotomy was perpendicular to the anatomical axis of the tibia. We used our original intraoperative protractor and an extramedullary guide.

### Preoperative planning

**Figure 1:**
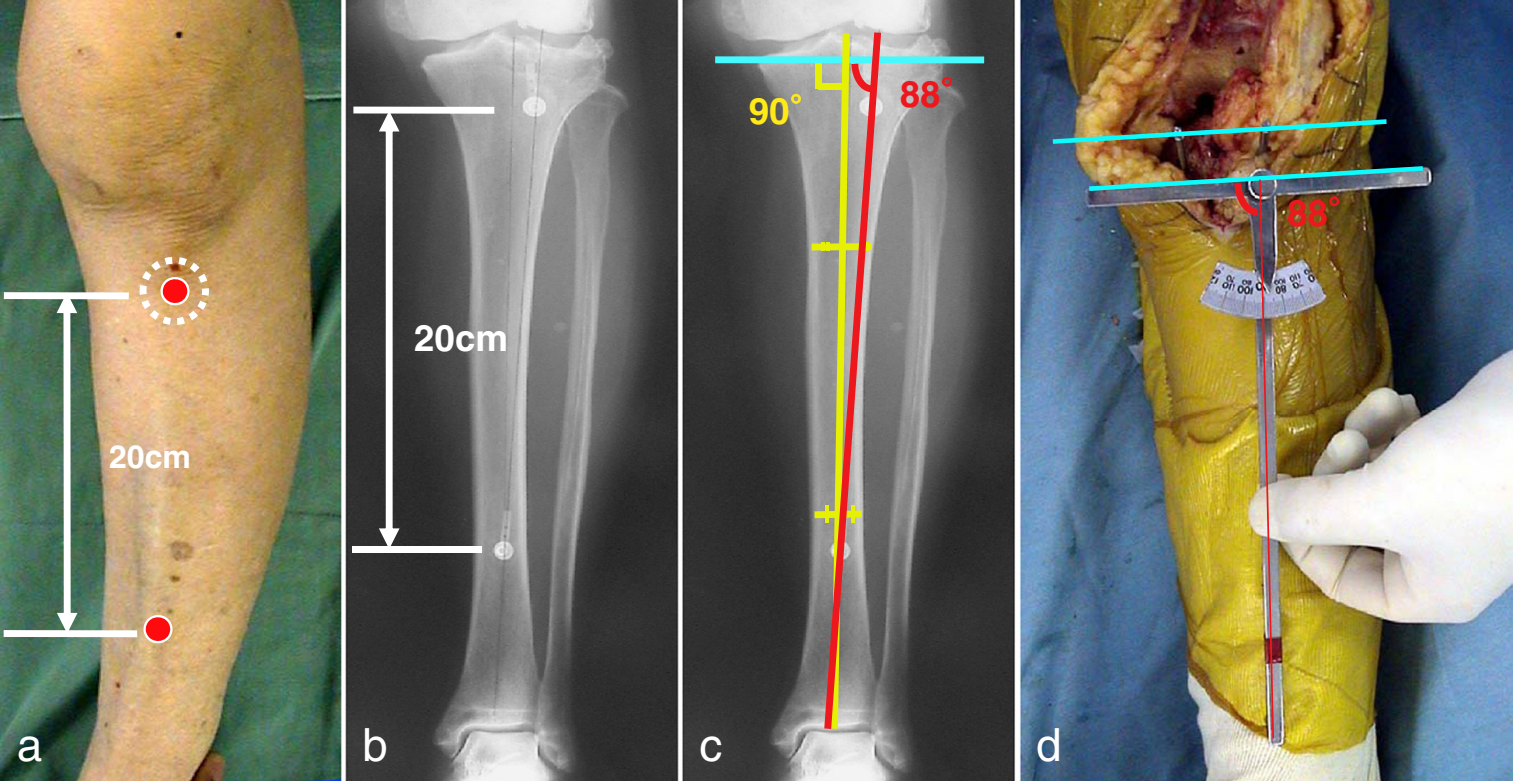
**Preoperative planning (a–c) and intraoperative protractor method (d).** (**a**) Two metallic markers are placed on the skin, one at the tibial tuberosity and the other at the anterior tibial crest. (**b**) The markers are 20 cm apart. Anteroposterior radiographs of the leg are obtained. (**c**) We defined the line going along the two markers as the tuberosity line (*red line*). The osteotomy line (*blue line*) is perpendicular to the anatomical axis of tibia (*yellow line*). We calculated the angle between the tuberosity line and the anatomical axis of tibia. Finally, we calculated the angle of the osteotomy line with the tuberosity line and called it the osteotomy angle. In this case, the angle of the osteotomy line with the tuberosity line is 88°. (**d**) The 20-cm bar is in accord with the tuberosity line, and the 10-cm bar is in synchrony with the osteotomy line. We set the osteotomy angle of the protractor based on a preoperative measurement. In this case, the protractor is set at 88°. We cut the bone parallel to the osteotomy line (*blue line*) determined by the protractor.

Two metallic markers were placed on the skin: one on the top of the tibial tuberosity and the other on the top of the anterior tibial crest (Figure 1a). The markers were 20 cm apart. These two points are easy to palpate from the surface of the body. Anteroposterior radiographs of the legs were obtained (Figure 1b). We constructed the angle of the osteotomy based on the X-ray films. We defined the line between the two markers as the tuberosity line. We defined the median line of the tibial shaft as the anatomical axis of the tibia in the coronal plane. The osteotomy line was perpendicular to the anatomical axis of the tibia. We then calculated the angle between the tuberosity line and anatomical axis of tibia. Finally, we calculated the angle of the osteotomy line with the tuberosity line and called it the osteotomy angle (Figure 1c).

### Intraoperative protractor method

**Figure 2:**
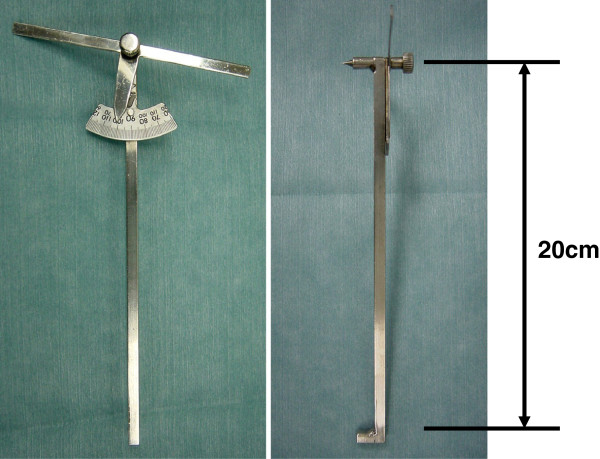
**View of our original intraoperative protractor (*right*, anteroposterior view; *left*, lateral view).** The protractor is constructed with a 10-cm bar and a 20-cm bar. There is a projection at a point of intersection where the 10-cm bar is at the tibial tuberosity and the distal 20-cm bar is at the anterior tibial crest. The angle made by the two bars make is displayed on a scale at their intersection.

**Figure 3:**
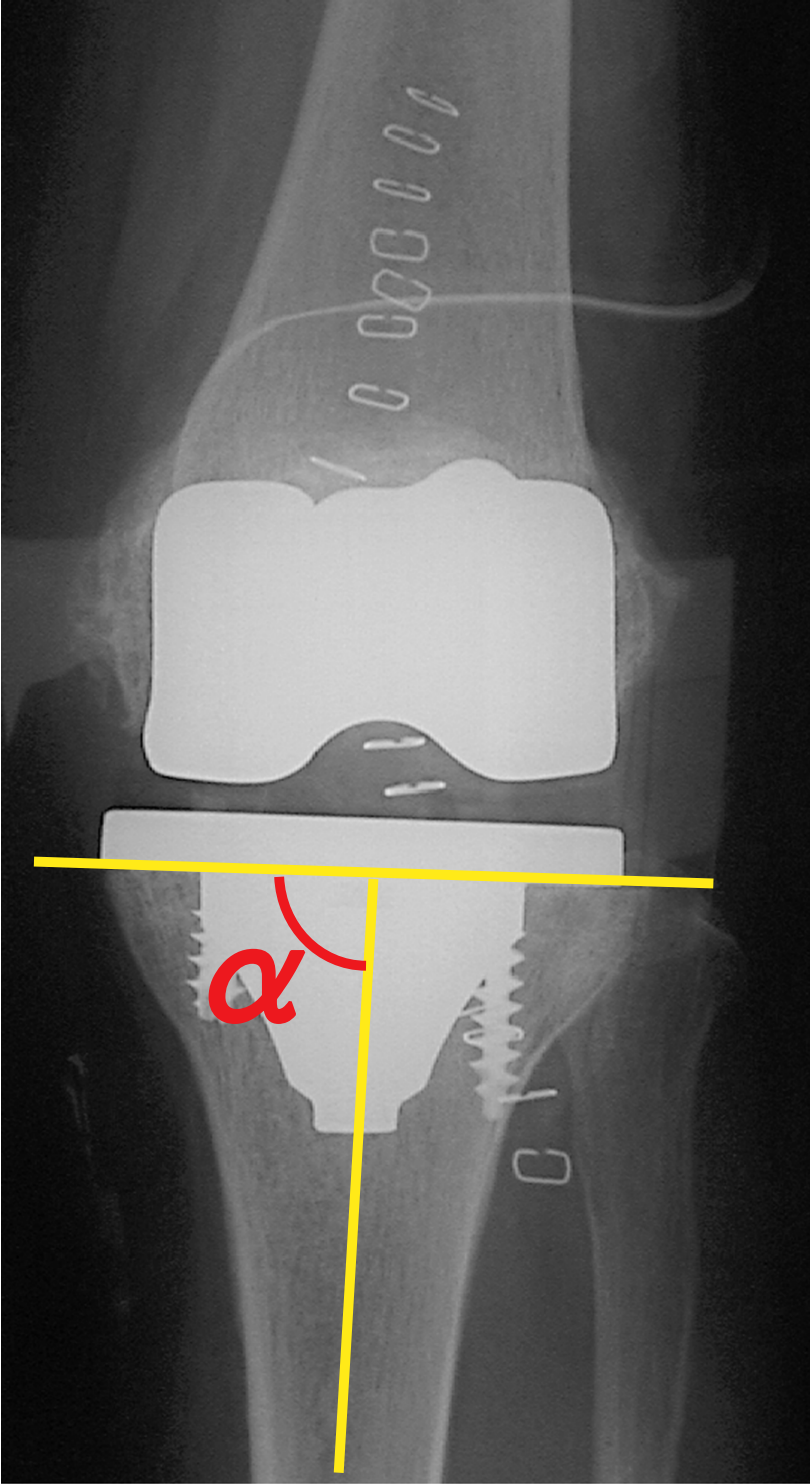
**Anteroposterior radiograph of the knee.** α: varus angle of the tibial osteotomy.

We made our original intraoperative protractor from stainless steel. The protractor is constructed with a 10-cm bar and a 20-cm bar. There is a point of intersection after one end is placed at the top of the tibial tuberosity and the distal 20 cm is at the top of the anterior tibial crest. Intraoperatively, we were able to confirm the two points easily. The angle made by the two bars is displayed on a scale at the intersection part (Figure 2). The 20-cm bar is in accord with the tuberosity line, and the 10-cm bar is in synchrony with the osteotomy line. We set the osteotomy angle of the protractor based on a preoperative measurement. We marked a line parallel to the protractor as the proximal tibia by marker or electric knife. We adjusted the extramedullary cutting guide parallel to the mark. We then cut the bone parallel to the osteotomy line of the protractor with a posterior slope of 5° in the sagittal plane (Figure 1d). The rotational alignment of the tibial component was adjusted to the anteroposterior axis between the center of the cut surface and the border of the medial third of the tibial tuberosity. Postoperatively, we analyzed the varus angle of the tibial osteotomy (Figure 3).

### Statistical analyses

The data for the varus angle of the tibial osteotomy was compared with the target angle (90°) using Student’s *t*-test, with *p* < 0.05 indicating significance.

## Results

The average varus angle of the tibial osteotomy using the protractor was 89.4° ± 1.6° [95% confidence interval (CI) was −1.0976, 0.0119]. There was no significantly difference from 90° (*p* = 0.055). The 90° and the 90° ± 2° tibial osteotomy varus angles were 42.9% and 82.9%, respectively.

## Discussion

The average varus angle of the tibial osteotomies using the protractor was 89.4° ± 1.6° (95% CI was −1.0976, 0.0119). The intraoperative protractor method is simple, and the accuracy is high. The tibial tuberosity and the tibial crest are easily palpated, so it is easy to identify the tuberosity line during the operation. The plasticity of the intraoperative tuberosity line can be high. The protractor can be used to confirm the cutting line after setting osteotomy jigs as an indicator. Recently, the use of a computer-assisted navigation system that achieved a high degree of accuracy relative to the desired target alignment has been described [9,10,14]. However, unpredictable complications, such as displaced or stress femoral or tibial fractures, have been reported to occur a few weeks after the operation with the use of a computer navigation system [15,16]. The protractor method is simple and low-priced, and the accuracy is high.

There were no complications using the protractor method. However, there are a few cautions to be noted. Anteroposterior radiography of the tibia is necessary before the operation. Also, the tibial tuberosity must be well palpated to reproduce the position of the metallic marker before the surgery: 5 mm away from the tibial tuberosity can make the error of 1.5° when performing the osteotomy. If there is deformity in the distal tibial crest where the marker has been placed, the tibial osteotomy may not be perpendicular to the tibial shaft. With these precautions, we found satisfactory accuracy for performing tibial osteotomy in the coronal plane using the protractor.

Regarding the limitation of this study, the present method was just 2-dimensional (2D), and accuracy of implant positioning was also performed with 2D radiographic analysis, which was far behind the current 3-dimensional (3D) tools, such as computed tomography (CT)-based navigation [9,10,14], 3D-preoperative planning software [11], and patient specific instrumentation system [12]. Therefore, a potential weakness of the study was the lack of spatial recognition. However, the present method without CT appears to have a merit in minimizing the risk of radiation exposure compared to the current tools. The study lacked a control group without the protractor, and we used small numbers of subjects. The comparative study between using the protractor and a conventional method using large numbers of subjects are necessary to clarify the effect of the protractor in the future.

## Competing interests

Both authors declare that they have no competing interests.

## Authors’ contributions

HM and KI conceived of the study and participated in its design and coordination. HM participated in the sequence alignment and drafted the manuscript. All authors read and approved the final manuscript.
